# Evaluation of Lasting Effects of Heat Stress on Sperm Profile and Oxidative Status of Ram Semen and Epididymal Sperm

**DOI:** 10.1155/2016/1687657

**Published:** 2016-01-17

**Authors:** Thais Rose dos Santos Hamilton, Camilla Mota Mendes, Letícia Signori de Castro, Patrícia Monken de Assis, Adriano Felipe Perez Siqueira, Juliana de Carvalho Delgado, Marcelo Demarchi Goissis, Teresa Muiño-Blanco, José Álvaro Cebrián-Pérez, Marcílio Nichi, José Antonio Visintin, Mayra Elena Ortiz D'Ávila Assumpção

**Affiliations:** ^1^Department of Animal Reproduction, School of Veterinary Medicine and Animal Science, University of Sao Paulo, Avenue Prof. Dr. Orlando Marques de Paiva 87, Cidade Universitária, 05508 270 Sao Paulo, SP, Brazil; ^2^Department of Biochemistry and Molecular and Cell Biology, University of Zaragoza, Miguel Servet 177, 50013 Zaragoza, Spain

## Abstract

Higher temperatures lead to an increase of testicular metabolism that results in spermatic damage. Oxidative stress is the main factor responsible for testicular damage caused by heat stress. The aim of this study was to evaluate lasting effects of heat stress on ejaculated sperm and immediate or long-term effects of heat stress on epididymal sperm. We observed decrease in motility and mass motility of ejaculated sperm, as well as an increase in the percentages of sperm showing major and minor defects, damaged plasma and acrosome membranes, and a decrease in the percentage of sperm with high mitochondrial membrane potential in the treated group until one spermatic cycle. An increased enzymatic activity of glutathione peroxidase and an increase of stressed cells were observed in ejaculated sperm of the treated group. A decrease in the percentage of epididymal sperm with high mitochondrial membrane potential was observed in the treated group. However, when comparing immediate and long-term effects, we observed an increase in the percentage of sperm with low mitochondrial membrane potential. In conclusion, testicular heat stress induced oxidative stress that led to rescuable alterations after one spermatic cycle in ejaculated sperm and also after 30 days in epididymal sperm.

## 1. Introduction

The testicle is the organ of the male reproductive tract responsible for spermatogenesis. In mammals, the testis temperature must range from 2 to 8°C below body temperature to ensure successful spermatogenesis [1]. The lower temperature is maintained by a cooling system comprising the scrotum, pampiniform plexus, and muscles [1]. Higher temperatures would lead to an increase of testicular metabolism without a corresponding increase in blood supply, resulting in local hypoxia and deleterious effects for the tissue [2, 3]. Moreover, similar to organ transplantation procedures, a phenomenon known as hypoxia-reperfusion injury may occur [3, 4]. In this condition, the oxidative imbalance may occur after the reestablishment of the normal temperature and tissue reperfusion. This situation has been described in studies where suppression of testicular function under heat stress led to a decrease in fertility in ruminants [5, 6], murine [7], and human affected by varicocele [8]. These studies concluded that oxidative stress is the main factor responsible for damage caused by heat stress.

Oxidative stress is defined as the damage caused to biomolecules by the imbalance between prooxidative molecules overlapping antioxidative molecules [9]. The increase in reactive oxygen species (ROS) or decrease in antioxidant levels could happen after heat stress; however, the exact mechanism is still unknown. The use of experimental models is important due to obvious impossibility of human experimentation. In this context, the ram would be an interesting model based on the ease of maintenance and genetic proximity with human [10].

Spermatozoa are very sensitive to oxidative damage due to the high levels of polyunsaturated fatty acids (PUFAs) in the plasma membrane [11]. In addition, the reduced cytoplasm limits the intracellular antioxidant levels [11]. Structure and function of the sperm membrane are affected by oxidative stress and this compromises fertilization [12]. For instance, loss of membrane fluidity causes a decrease in sperm motility and impairs sperm-oocyte fusion [13, 14]. ROS not only affect the sperm membrane, as high levels in seminal plasma were negatively correlated with sperm motility and positively correlated with the incidence of sperm DNA fragmentation in infertile men [15]. Ram spermatozoa produce high levels of hydrogen peroxide, especially due to high amounts of polyunsaturated/saturated fatty acids and low proportions of cholesterol/phospholipids in the plasma membrane when compared with other species [16]. These ratios are responsible for an increased susceptibility to oxidative damage in the presence of ROS, and subsequent loss of membrane and acrosome integrity [16].

A variety of enzymatic and nonenzymatic antioxidants present in the plasma allows neutralization of ROS. Indeed, studies on lipid peroxidation and antioxidant enzymes in fertile and infertile men have shown an increase in superoxide dismutase (SOD) activity [17]. The epididymis is an important source for the antioxidant content in the seminal plasma, protecting sperm cell from oxidative damage during storage [18]. In this context, the study of the antioxidant activity in the epididymal environment could bring information about protection mechanisms after oxidative stress induced by heat stress. The relationship between spermatic attributes and the antioxidant activity present in the seminal plasma or in the sperm cell under oxidative stress conditions is poorly described in ovine.

The antioxidant response to a stressful event may involve an immediate response in cases of acute situations, accomplished mainly by protein activation. On the other hand, a long-term response is also important, which would require gene activation and translation of new proteins [19, 20]. Little is known about the contribution of the epididymis to the maintenance of oxidative balance in immediate or long-term response to stressful conditions. The dynamics of antioxidant responses are critical during spermatogenesis once it is a cyclical and continuous event. Therefore, when considering the testicular and epididymal environments, the longitudinal effect of this oxidative balance must be performed considering the sperm cycle.

In this context, the aim of this study was to evaluate how heat stress, induced by ram testicular insulation, affects sperm profile and the enzymatic antioxidant activity in ejaculated sperm during consecutive weeks or in epididymal sperm immediately after insulation or in a long-term response.

## 2. Material and Methods

Unless otherwise indicated, all chemicals were obtained from Sigma Chemicals (St. Louis, MO). All experiments were performed using fresh ram semen collected using an artificial vagina. Semen collections were performed weekly, during nine weeks, from twelve mature (8 months old) Santa Ines rams. Animals belonged to the Department of Animal Reproduction of the School of Veterinary Medicine and Animal Science from the University of Sao Paulo. The animals were submitted to uniform nutritional conditions, and the experiments were approved by the Bioethics Committee of the School of Veterinary Medicine and Animal Sciences, University of Sao Paulo (protocol number 2445-2011).

### 2.1. Reagents and Solutions

All chemical reagents and solutions used in this study were purchased from Sigma-Aldrich (St. Louis, MO, EUA) unless otherwise stated.

### 2.2. Experiment  1: Lasting Effects of Heat Stress on Sperm Profile and Oxidative Status on Ejaculated Sperm

The animals were randomly divided into two groups: animals undergoing testicular insulation (treated, *n* = 6) and control group (*n* = 6). An insulating bag was placed in the testicles of animals from the treated group to induce heat stress effects on spermatogenesis. The bags were kept for 288 consecutive hours, and during this period the internal temperature of each bag and environmental temperature were monitored using a digital thermometer. After the removal of the bags, semen was collected weekly, during 9 weeks.

#### 2.2.1. Immediate and Morphological Sperm Evaluations

The following evaluations were immediately performed: seminal volume (mL), motility (%), and mass motility (0–5). Sperm concentration count was performed using a hemocytometer. Sperm morphological abnormalities were assessed in a phase contrast microscope at 1000x magnification under oil using 10 *μ*L of fresh semen fixed in 1 mL of buffered formalin (Phosphate-Buffered Saline, PBS, Gibco, Life Technologies, Carlsbad, USA, with 2% of formalin 37%). Sperm abnormalities were quantified and classified into major and minor defects, and the sum of defects was considered as total defects [21].

A total of 200 cells per sample were evaluated.

#### 2.2.2. Flow Cytometry (Plasma and Acrosome Membranes Integrity, Mitochondrial Membrane Potential, and Oxidative Status)

Plasma membrane and acrosome integrities were evaluated by propidium iodide (PI) and fluorescein isothiocyanate-conjugated* Pisum sativum* agglutinin (FITC-PSA), respectively. The association of these fluorescent probes divides sperm populations into four groups: intact membrane and intact acrosome (IMIA), intact membrane and damaged acrosome (IMDA), damaged membrane and intact acrosome (DMIA), and damaged membrane and damaged acrosome (DMDA). The procedure was performed with 200,000 cells diluted in SP-Talp [Modified Tyrode's Albumin Lactate Pyruvate (NaCL 0.1 M, KCl 0.003 M, MgCl_2_ 0.0004 M, NaH_2_PO_4_ 0.0003 M, NaHCO_3_ 0.025 M, CaCl_2_H_2_O 0.003 M, Ácido Lático Syr 0.3% v/v, Hepes 0.01 M, pH 7.4, and Osm 295–300)] and stained for 5 minutes with 0.5 mg/mL PI and 100 *μ*g/mL FITC-PSA. Samples were analyzed by flow cytometry, using a 488 nm excitation laser and emission was detected at 630–650 nm (PI) and 515–530 nm (FITC). Mitochondrial membrane potential was evaluated using JC-1 probe (5,5′,6,6′-tetrachloro-1,1′,3,3′-tetraethyl-benzimidazolylcarbocyanine chloride, Invitrogen, Eugene, OR, USA). This probe emits green fluorescent light from cells with low (LMM) and medium (MMM) mitochondrial potential or red-orange fluorescent from cells with high mitochondrial potential (HMP). The procedure was performed with 200,000 cells diluted in SP-Talp and stained with 76.5 *μ*M JC-1 for 5 minutes. Samples were analyzed by flow cytometry, excited at 488 nm, and detected at 590 nm. Sperm oxidative stress was assessed using fluorescent probe dichlorofluorescein (2′,7′-dichlorofluorescein diacetate, DCF). DCF emits fluorescence when in contact with free radicals [14, 22, 23]. Evaluations were performed by flow cytometry. In brief, approximately 4,000 spermatozoa were resuspended in 1 *μ*L of TALP medium and incubated with 3.5 *μ*L of 1 mM fluorescent probe DCF for 5 minutes. Propidium iodide (PI; 0.5 mg/mL, 0.5 *μ*L) was added simultaneously to identify and exclude cells with damaged membrane, as this condition does not allow proper function of DCF stain. Flow cytometer analysis was performed as described above using the 525 nm detector (green fluorescence). The same protocol was performed in Experiment  2.

Flow cytometry analysis of sperm samples was performed using Guava EasyCyte Mini System (Guava Technologies, Hayward, CA, USA). A total of 10,000 events per sample were analyzed and data corresponding to yellow (PM1 photodetector—583 nm), red (PM2 photodetector—680 nm), and green (PM3 photodetector—525 nm) fluorescent signals were recorded after a logarithmic amplification. For analysis, cell doublets and debris were excluded using PM3/FSC (forward scatter). All data was analyzed by FlowJo version 8.7 software.

#### 2.2.3. Seminal Plasma Enzymatic Activity

Seminal plasma was obtained by centrifuging 500 *μ*L of fresh semen at 5°C for 10 minutes at 660 g. The supernatant was recovered and stored at −20°C for further analysis. Measurements were performed based on the rate of substrate consumption in reactions catalyzed by each antioxidant enzyme in a given time interval using a spectrophotometer (Evolution 300 UV-Vis, Thermo Scientific, Waltham, MA, USA). Activities of superoxide dismutase (SOD), glutathione peroxidase (GPx), and glutathione reductase (GRD) were determined as described previously [24]. In addition, catalase activity was determined by evaluating the consumption of hydrogen peroxide for 3 minutes at 242 nm, and the 18.6 × 10^3^ cm^−1 ^M^−1^ molar extinction coefficient was used.

#### 2.2.4. Quantification of Antioxidant Enzymes

We used SDS-polyacrylamide gel electrophoresis and western blotting to quantify the levels of antioxidant enzymes in the seminal plasma. Total protein concentration (Protein Assay, Bio-Rad, Hercules, CA, USA) in seminal plasma was determined by the Bradford method [25]. Then, 20 mg of protein was mixed with 5 *μ*L of loading buffer (0.045 M Tris/HCl, 0.8 mM EDTA, 3% SDS 10% glycerol, 5% *β*-mercaptoethanol, and 0.004% bromophenol blue) and loaded into wells. Proteins were separated by dimension on 12% polyacrylamide gel (v/v) by standard SDS-PAGE using a Mini Protean III System (Bio-Rad, Hercules, CA, USA). A mixture of prestained protein standards was used as marker, with molecular weights ranging from 10 to 250 kDa (Bio-Rad, Hercules, CA, USA). Electrophoresis was performed for 90 minutes at 130 V at 4°C. Subsequently, proteins were blotted onto polyvinylidene difluoride (PVDF) membranes using the Trans-Blot Turbo for 10 minutes at 2.5 A, 25 V (Bio-Rad, Hercules, CA, USA). After air-drying the membrane, blocking of nonspecific sites was performed with 5% BSA in PBS for 2 hours. Membranes were incubated overnight at 4°C with primary antibodies; anticatalase (SC 50508, H-300, Santa Cruz Biotechnology, Dallas, Texas, USA), anti-SOD (SOD-3, SC 67088, H-90, Santa Cruz Biotechnology), anti-GPx (GPx-5, SC 50498, H-45, Santa Cruz Biotechnology), and anti-GRD (antiglutathione reductase antibody ab84963, Abcam, Cambridge, Fl, UK) diluted in 1/1000 PBS-Tween with 1% BSA. After 3 washes every 5 minutes, membranes were incubated with FITC conjugated secondary donkey anti-rabbit antibody (Li-COR Biotechnology, Bad Homburg, Germany, 1/15000 in PBS-Tween with 1% BSA) for 75 minutes at room temperature and protected from light. Quantifications of signal intensities and areas of bands were performed by scanning the membranes using Odyssey CLX (Li-Cor Biotechnology, Bad Homburg, Germany). Results were expressed considering the relationship between signal (pixel) and band area.

#### 2.2.5. Measurement of Seminal Plasma (Spontaneous) and Sperm (Induced) Thiobarbituric Acid Reactive Substances (TBARS)

This technique is based on methodology previously described by Ohkawa et al. [26], in which two thiobarbituric acid molecules reacted with one molecule of malondialdehyde, producing a pink color complex, which was measured spectrophotometrically at 532 nm. This reaction occurs between 90 and 100°C at an acidic pH. For the determination of spontaneous TBARS in seminal plasma, aliquots of 300 *μ*L of fresh semen associated with 600 *μ*L of 10% trichloroacetic acid solution were centrifuged at 5°C, 16,000 g, for 10 minutes to precipitate the proteins. After precipitation of proteins, approximately 700 *μ*L of the supernatant was frozen at −20°C for further analysis. For quantification of induced TBARS, about 1 million sperm diluted in 200 *μ*L of PBS were incubated with 4 mM of ferrous sulfate (50 *μ*L) and 20 mM ascorbate (50 *μ*L) at 37°C for 1.5 hours, as described by Simões et al. [27]. Immediately after ROS induction, 600 *μ*L of 10% (v/v) trichloroacetic was added to the mixture (2 : 1) in order to precipitate proteins and cellular debris. Samples were centrifuged (16,000 g, for 10 minutes) and the supernatant was recovered (500 *μ*L) and stored (−20°C). TBARS seminal and sperm samples were thawed and incubated with 500 *μ*L of a 1% thiobarbituric acid solution (in NaOH 0.05 M) for 10 minutes at 90–100°C. Reaction was stopped by placing samples on ice. Levels of TBARS were assessed using a spectrophotometer at 532 nm. Results were compared to a standard curve previously prepared with malondialdehyde. Malondialdehyde is the major substance that reacts with thiobarbituric acid, and the TBARS concentration was determined using the value of 1.56 × 10^5^ M^−1^ × mL^−1^ as the malondialdehyde molar extinction coefficient. Lipid peroxidation in semen is expressed in TBARS/mL nanograms of seminal plasma (spontaneous) or for each 10^6^ sperm (induced). The same protocol for induced TBARS was performed in Experiment 2.

### 2.3. Experiment  2: Study of Immediate and Long-Term Effects of Heat Stress on Epididymal Sperm

The same animals distributed in the same experimental groups were subjected to a second period of testicular insulation, which lasted 10 days (240 hours). This was performed 60 days after the end of the first experiment when the sperm profile returned to results similar to those observed in the beginning of the first experiment. The early and the late effect of heat stress on epididymal sperm were evaluated in this study. Thus, each animal was subjected to two unilateral orchiectomies: the first one 24 hours after the removal of insulation bag (D0) and the second one in 30 days after the first orchiectomy (D30).

#### 2.3.1. Epididymis Sperm Collection

After surgery, epididymides were immediately taken to the laboratory and washed in saline solution at 37°C. To collect epididymis semen samples, small incisions were performed with a scalpel blade in the epididymis tail, and pressure was applied on its base using hemostats and sperm collected with the aid of automatic pipette [28]. Sperm were resuspended in PBS for sperm concentration assessment.

#### 2.3.2. Computer Assisted Sperm Analysis (CASA) System

Motility parameters of epididymis semen were performed by Computer Assisted Sperm Analysis System (CASA, Hamilton Thorne IVOS). The chambers (Standard Count 4-chamber slide, 20 microns, Leja), heated at 37°C, were filled with 6 *μ*L of sample (approximately 5 × 10^7^ cells/mL), and 5 fields were selected for the analysis, in which approximately 1 × 10^6^ sperm cells were analyzed. The setup used was as follows: image capture: frames per sec = 60 Hz, and number of frames = 45; cell detection: minimum contrast = 70, minimum cell size = 5 pix; defaults: cell size = 10 pix, cell intensity = 80; progressive cells: path velocity (VAP) = 50 *μ*/s, straightness (STR) = 80%; slow cells: VAP cutoff = 20 *μ*/s, VSL cutoff = 5 *μ*/s; static intensity gates = minimum 0.20 and maximum 1.92; static intensity gates = minimum 0.60 and maximum 4.32; and static elongation gates = minimum 7 and maximum 91. The following parameters were evaluated: mean average velocity (VAP, *μ*m/s), curvilinear velocity (VCL, *μ*m/s), straight-line velocity (VSL, *μ*m/s), linearity coefficient (LIN, %), straightness coefficient (STR, %), amplitude of lateral head displacement (ALH, *μ*m), beating cross frequency (BCF, Hz), total (%) and progressive (%) motility, and percentage of cells with fast, medium, slow, and static movement.

#### 2.3.3. Epididymal Sperm Enzymatic Activity

After extraction, epididymal sperm were diluted in BotuBov (Botupharma Animal Biotechnology, Botucatu-SP, Brazil) and frozen at −196°C. Standard freezing curve was performed (37°C to 5°C in 2 hours with a decrease of −0.25°C/min; 3 hours and 45 minutes in balance time, −20°C with a decrease of −5°C/min, −196°C with a decrease of 125°C/min). Straws were thawed at 37°C (90 seconds) and removal of the diluent was performed by addition of 1 mL of semen diluted in SP-Talp (750 *μ*L of semen in 3 mL of SP-Talp) carefully on 7.5 mL of sucrose solution (0.9% NaCl, 7.5% sucrose, and 0.18% glucose). After two centrifugations (200 g/5 minutes and 900 g/10 min), the supernatant was discarded and 1 mL of the sediment was incubated with 200 *μ*L 4% Triton during 30 minutes in water bath with agitation. Samples were centrifuged (600 g/8 minutes), and the supernatant was removed and stored at −20°C for further analysis. Quantification of intracellular antioxidant activity of SOD and GPx enzymes was performed according to Nichi et al. [5]. SOD activity was measured indirectly by reduction of cytochrome c by superoxide (O_2_^−^) generated by xanthine oxidase/xanthine system. The SOD present in the sample competes with cytochrome c by converting superoxide into hydrogen peroxide. We observed absorbance for 5 minutes in spectrophotometer at 470 nm at 25°C. Enzymatic activity of GPx was based on the consumption of NADPH by GSSH conversion into GSH. A reaction was induced between hydrogen peroxide and reduced glutathione (GSH), catalyzed by GPx and the enzyme glutathione reductase (GSR). NADPH consumption was detected at a wavelength of 340 nm, for 10 minutes at 37°C (measurements at every 5 seconds). SOD and GPx activity results were expressed in IU/10^6^ sperm. Molar attenuation coefficient of NADPH (6.22 mM^−1 ^cm^−1^) was used for determination of values.

## 3. Statistical Analysis

The dependent variables were analyzed by Statistical Analysis System  9.3 (SAS Institute, Cary, NC). All data were tested for normality of residues and homogeneity of variance. Variables that did not comply with these statistical premises were subjected to transformations. In Experiment 1, the MIXED procedure was used for analysis of variance with repeated measures over time. Comparisons of means were performed using* least square means* (LS means) for different dependent variables and for each condition of the statistical model (treatment, week, and treatment × week). For nonparametric data, we used nonparametric analysis of variance (Kruskal Wallis, through NPAR1WAY procedure), and for comparison of means we used comparison between two groups at a time (Wilcoxon). The parametric results are presented as mean ± standard error. The nonparametric results are presented as median (low quartile, high quartile). In Experiment 2, the analysis of variance was carried using GLM procedure considering the 2 × 2 factorial. Factors considered treatment effect (treated group versus control group), and immediate and long-term effect of the damage induced by heat stress (first and second unilateral orchiectomies), with subsequent comparison of means by the LSD method. A 5% significance level was used to reject the hypothesis of nullity.

## 4. Results

### 4.1. Experiment  1

#### 4.1.1. Testicular and Environment Temperature

The mean internal temperature was 33.29°C ± 0.34 for treated and 28.05°C ± 1.30 for control group. The mean environment temperature and relative humidity in this period were 17.01°C ± 0.52 and 78.08% ± 0.73, respectively.

#### 4.1.2. Immediate Evaluation and Sperm Morphology

**Figure 1:**
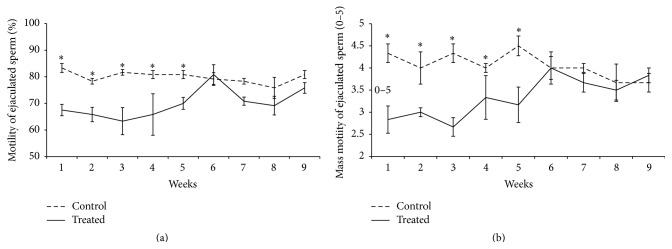
Comparison of motility (a) and mass motility (b) of ejaculated sperm considering the interaction effect between treatment and week in rams submitted or not to heat stress. The results are presented as means ± SEM. Asterisk represents significant differences (*p* < 0.05).

**Figure 2:**
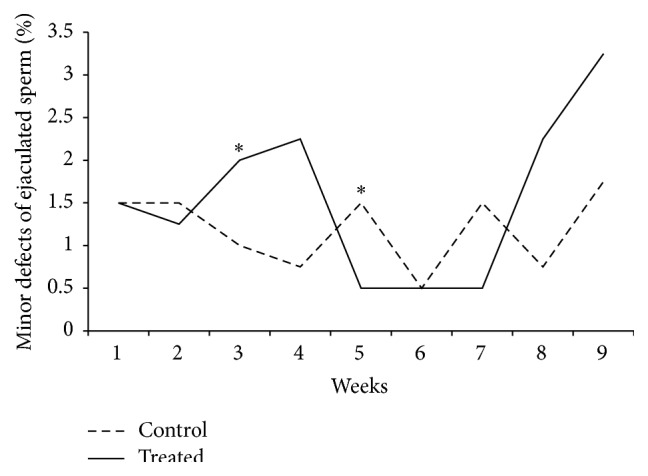
Percentages of minor defects observed in ejaculated sperm considering the interaction effect between treatment and week in rams submitted or not to heat stress. Graphic presented as mean. Asterisk represents significant differences (*p* < 0.05). T: treated, C: control.

An interaction between treatment and week of sample collection was observed for the variables motility (*p* = 0.007, Figure 1(a)), mass motility (*p* = 0.0001, Figure 1(b)), and percentage of sperm with minor defects (*p* < 0.05). Considering motility and mass motility, a decrease in the percentage of mobile cells was verified in the treated group until the fifth experimental week (Figures 1(a) and 1(b)), one spermatic cycle. From the sixth week onwards, differences between groups were no longer observed, which may indicate a recovery response to heat stress. In regard to minor defects, the treated group presented higher percentages of defects when compared to the control group in the fourth experimental week (Figure 2). In regard to sperm major defects, a treatment effect was observed, as a significant increase in the number of defects was detected in the treated group [2.5 (1.5; 4.5)] when compared to the control [2 (1.5; 3)]. Media values, standard errors, and *p* values of all variable measured to immediate evaluation and sperm morphology for treatment, week, and interaction effect are present in Supplementary Material (see Tables  S1, S2, and S3 in Supplementary Material available online at http://dx.doi.org/10.1155/2016/1687657).

#### 4.1.3. Evaluation of Plasma and Acrosome Membranes Integrity, Mitochondrial Membrane Potential, and Intracellular Marking of Free Radicals in Sperm

**Figure 3:**
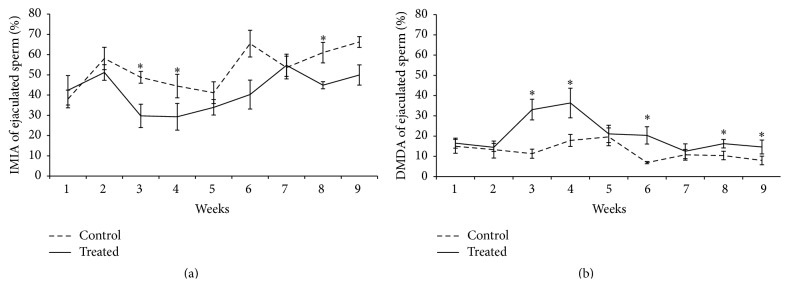
Percentages of IMIA (a) and DMDA (b) of ejaculated sperm considering the interaction effect between treatment and week in rams submitted or not to heat stress. Graphic presented as means ± SEM. Asterisk represents significant differences (*p* < 0.05). IMIA: intact membrane and intact acrosome sperm, DMDA: damaged membrane and damaged acrosome sperm.

**Figure 4:**
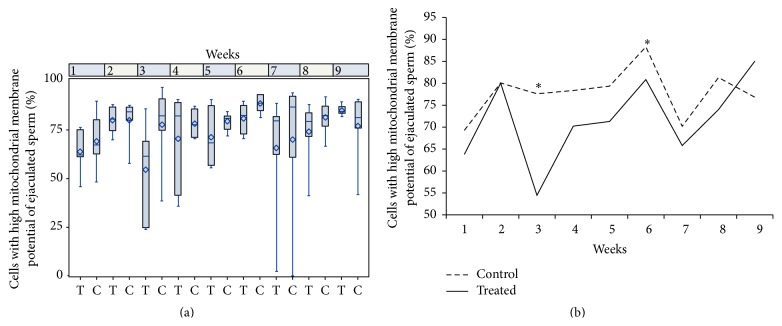
Percentages of cells with high mitochondrial membrane potential of ejaculated sperm considering the interaction effect between treatment and week in rams submitted or not to heat stress. (a) Boxplot presented as median (superior quartile, inferior quartile). (b) Data presented as mean. Asterisk represents significant differences (*p* < 0.05). T: treated, C: control.

**Figure 5:**
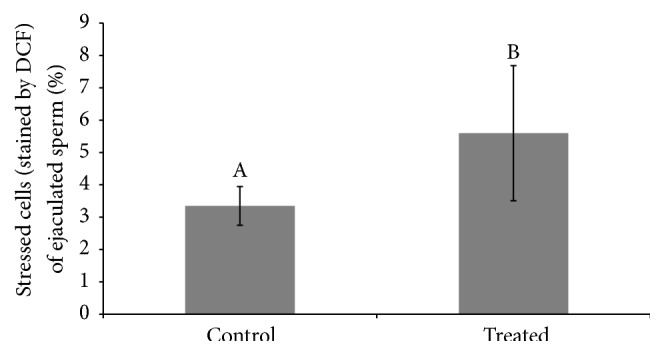
Percentage of stressed cells (stained by dichlorofluorescein, DCF) of ejaculated sperm considering the treatment effect between treated and control group in rams submitted or not to heat stress. Data presented as means ± SEM. Different superscript letters in each bar represent significant differences (*p* < 0.05).

An interaction between treatment and week of sample collection was observed in the percentage of IMIA (*p* = 0.028, Figure 3(a)) and DMDA (*p* < 0.05, Figure 3(b)). The treated group presented an increase in the percentage of DMDA sperm compared with control group during the first sixth experimental weeks. We also verified a reduction in the percentage of IMIA sperm in the treated group too in the same experimental weeks. Considering the percentage of sperm only with acrosome damage (IMDA), treatment effects and interactions were not observed, while effect of time was present (weeks, Table S2). It is noteworthy that this IMDA percentage was higher in the seventh week when compared to the other weeks. Concerning the percentage of sperm only with membrane damage (DMIA, Table S2), interaction and treatment effects were not observed, while effect of time was present when the treated group presented higher percentages at the sixth week when compared to others. In regard to mitochondrial membrane potential, an interaction was observed. Treated group showed lower percentages of cells with high mitochondrial membrane potential at weeks 3 and 6 when compared to control (Figures 4(a) and 4(b)). An increase in the percentage of cells displaying oxidative stress (stained by dichlorofluorescein) was observed in the treated group when compared to the control group (5.60 ± 2.09 versus 3.35 ± 0.6, resp., Figure 5). Media values, standard errors, and *p* values of all variables measured to plasma and acrosome membranes integrity, mitochondrial membrane potential, and intracellular marking of free radicals in semen sperm for treatment, week, and interaction effect are present in Supplementary Material (Tables  S1, S2, and S3).

#### 4.1.4. Enzymatic Activity and Western-Blot in Seminal Plasma

**Figure 6:**
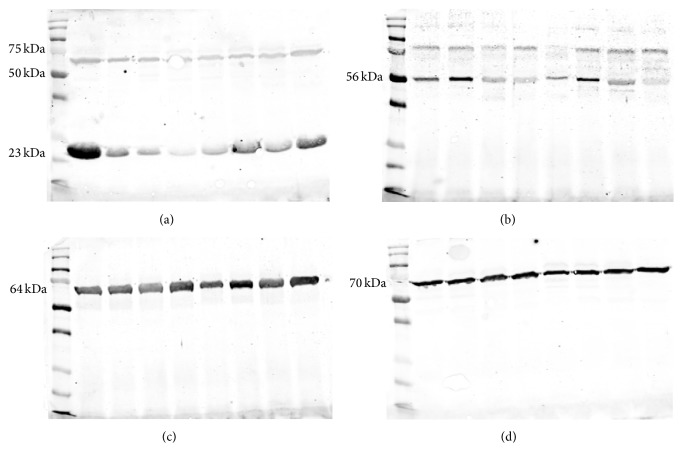
Immunoblotting detection of glutathione peroxidase, GPx (a), glutathione reductase, GDR (b), catalase (c), and superoxide dismutase, SOD (d), in seminal plasma of ejaculated sperm in rams submitted or not to heat stress.

**Figure 7:**
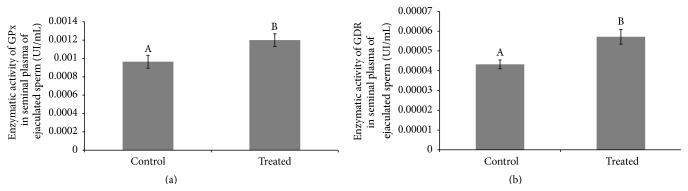
Comparison of enzymatic activity of glutathione peroxidase, GPx (a), and glutathione reductase, GDR (b), in seminal plasma of ejaculated sperm considering the treatment effect between treated and control group in rams submitted or not to heat stress. Graphic presented as means ± SEM. Different superscript letters in each bar represent significant differences (*p* < 0.05).

We observed one protein band of 23 kDa and one between 50 and 75 kDa (Figure 6(a)) corresponding to GPxBI and GPxBS, respectively [29, 30]. GDR exhibited one strong protein band of 56 kDa (Figure 6(b)), catalase showed one protein band of 64 kDa (Figure 6(c)), and SOD presented one protein band of 70 kDa (Figure 6(d)). An increase in the enzymatic activity of GPx and GRD was detected in the treated group when compared to the control (GPx: 0.00120 ± 0.000069 UI/mL versus 0.00096 ± 0.000070 UI/mL, Figure 7(a); GRD: 0.000081 ± 0.0000048 UI/mL versus 0.000043 ± 0.00000221 UI/mL, Figure 7(b)). No differences were observed between SOD and catalase enzymatic activities. Quantification of enzymes through western blotting did not allow observation of interaction or treatment effects for GPx, GRD, SOD, and catalase. We observed an effect of time when quantifying GPx (GPxBI *p* = 0.003, GPxBS *p* = 0.002) and catalase (*p* < 0.001) (Table S2). Media values, standard errors, and *p* values of all variables measured to enzymatic activity and western-blot in seminal plasma for treatment, week, and interaction effect are present in Supplementary Material (Tables S1, S2, and S3).

#### 4.1.5. Lipid Peroxidation in Seminal Plasma and Sperm

No differences on TBARS levels in seminal plasma were observed between treated and control groups. However, an effect of week for quantification of TBARS in sperm cell (*p* < 0.0001) and seminal plasma (*p* < 0.0001) was observed (Table S2). Media values, standard errors, and *p* values of all variables measured to enzymatic activity and western-blot in seminal plasma for treatment, week, and interaction effect are present in Supplementary Material (Tables S1, S2, and S3).

### 4.2. Experiment  2

#### 4.2.1. Computerized Motility Analysis

No differences in any of the CASA parameters measured were observed between treatments or in immediate and long-term effects of heat stress in the testicle (treatment, week, and interaction effect are present in Supplementary Material, Tables S4, S5, and S6).

#### 4.2.2. Evaluation of Plasma and Acrosomal Membranes Integrity, Mitochondrial Membrane Potential, and Intracellular Marking of Free Radicals in the Epididymal Sperm

**Figure 8:**
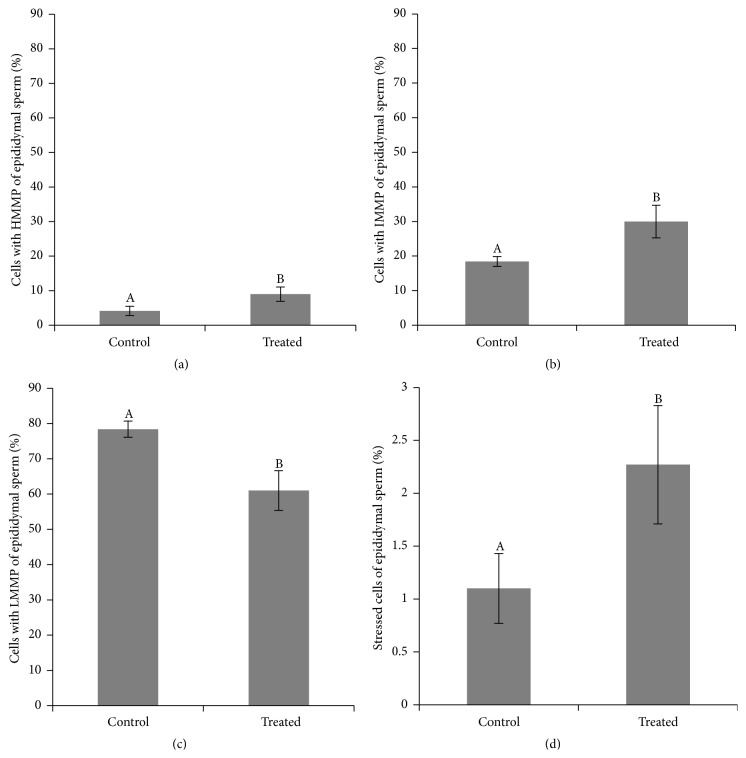
Comparison of percentage of high, HMMP (a), intermediary, IMMP (b), low, LMMP (c), mitochondrial membrane potential and percentage of stressed cells (stained by dichlorofluorescein (d)) of epididymal sperm considering the treatment effect between treated and control groups in rams submitted or not to heat stress. Graphic presented as means ± SEM. Different superscript letters in each bar represent significant differences (*p* < 0.05).

No differences were observed between groups and between immediate and long-term effects of heat stress on epididymal sperm in any categories of plasma and acrosome membranes integrity (treatment, week, and interaction effect are present in Supplementary Material, Tables S4, S5, and S6). Nonetheless, the treated group displayed a decrease in the percentage of cells with high mitochondrial membrane potential (61 ± 5.62% versus 78.39 ± 2.29%, Figure 8(a)). Furthermore, the treated group presented an increase in the percentage of cells with intermediary (29.96 ± 4.71% versus 18.41 ± 1.45%, Figure 8(b)) and low (9.04 ± 2.06% versus 4.15 ± 1.35%, Figure 8(c)) mitochondrial membrane potential, when compared to the control. No differences were observed between immediate and long-term effects of heat stress in high and intermediate mitochondrial membrane potential categories. We observed a decrease in low mitochondrial membrane potential in epididymal sperm considering the long-term response compared to immediate response to heat stress (2.75 ± 1.16% versus 10.44 ± 176%). Considering oxidative stress, there was an increase in the percentage of cells positive for DCF staining in the treated group when compared to the control (2.27 ± 0.56% versus 1.10 ± 0.33%, Figure 8(d)). No differences were observed between immediate and long-term effects of heat stress in stressed cells stained by DCF.

#### 4.2.3. Intracellular Enzymatic Activity and Lipid Peroxidation in Epididymal Sperm

No differences were observed between groups and between immediate and long-term effects of heat stress considering GPx and SOD activity in epididymal sperm (treatment, week, and interaction effect are present in Supplementary Material, Tables S4, S5, and S6). Regarding lipid peroxidation, no differences were observed between groups (control versus treated), and between immediate and long-term effects of heat stress (treatment, week, and interaction effect are present in Supplementary Material, Tables S4, S5, and S6) when considering TBARS.

## 5. Discussion

Heat stress has been related to decrease in sperm motility, concentration, and viability in mice [31], bull [5, 32–34], men [35], and ram [36, 37]. In our study, we observed a decrease in motility, vigor, and mass motility and an increase in the percentage of cells with major and minor defects up to the fifth experimental week. Similar results were observed when rams were submitted to heat stress for 14 or 28 days [38, 39]. Also, in the present study, acrosome and plasma membrane damage were observed in the sperm from the ejaculate after induced heat stress. In this case, the heat stress impaired sperm quality for approximately 1 sperm cycle (47 days). From the sixth experimental week onwards, no more differences were observed between groups for these variables, indicating a recovery of damage possibly caused by heat stress.

Lipids such as PUFAs are the most susceptible molecules to peroxidation in the sperm plasma membrane [40]. Peroxidation of PUFAs has been associated as the main cause of decrease in sperm motility due to the increase of ROS concentrations [41–44]. The increase of ROS has also been associated with sperm morphological alterations and teratospermia [44]. The extent of the damage depends on the nature and quantity of ROS involved, duration of exposure, and extracellular factors such as temperature and oxygen tension [40]. In our study, despite differences observed in sperm from the ejaculate, no differences were observed in epididymal sperm after the second testicular insulation. It is possible that cells susceptible to ROS damage were eliminated after the stress from the first insulation (Experiment  1), while cells more resistant to heat stress remained in the second induction (Experiment  2). These cells seem to have passed undamaged, with no alterations in motility and plasma and acrosome membrane integrity. Similar results were found in studies of renal patients, where the highest degree of apoptosis correlated with improved renal function six months after kidney transplant [45]. Apoptosis could be a mechanism involved in the elimination of susceptible cells [45].

In our study, heat stress treatment effectively increased ROS levels in both experiments as observed by DCF staining [23, 46, 47]. Furthermore, although a significant difference cannot be verified, there was a fourfold increase (8.89 ± 0.42% in the treated group versus 2.05 ± 3.42% in the control group, *p* = 0.24) in cell susceptibility to oxidative stress, quantified by induced TBARS. This evidence corroborates the idea that heat stress damage to the spermatic cell is mediated by oxidative stress. Regarding oxidative status evaluations, no difference was observed in TBARS quantification in seminal plasma; however, an increase of enzymatic activity of GPx and GRD was observed in the treated group when compared to the control. Some studies have correlated increases in GPx activity to situations in which oxidative stress is the reason of the pathological processes, such as in women with preeclampsia [48], patients with Down Syndrome [49], postexercise stress [50, 51], and even infertility [52, 53]. A study found a tenfold increase in GPx activity in infertile men compared to fertile ones [53], indicating that this enzyme may serve as a marker of oxidative imbalance. The increase of GPx activity would be a response to the increase of ROS, in particular, hydrogen peroxide [54].

Despite the increase in the enzymatic activity of GPx in seminal plasma, no differences were observed in the immunodetection. One hypothesis could be that GPx is present in the epididymis in an inactive state, becoming active when necessary [9]. In epididymal environment, this activation mechanism would be essential for sperm protection, since activation of already translated antioxidant enzymes would be faster and more effective than the synthesis of new ones, preventing oxidation reactions faster. Supporting this hypothesis, GPx activity was correlated with seminal characteristics in infertile men but it was not correlated to mRNA levels of GPx [54]. According to these authors, there is a posttranscriptional control of GPx activity, but the mechanisms are still unknown. Further studies are necessary to clarify how the activation of these antioxidant enzymes occurs in the seminal plasma. In epididymal environment, there were no differences between groups and between immediate and long-term responses to antioxidant enzyme activity quantified in sperm cells.

In this work, in both sperm from the ejaculate and epididymal sperm, there was a decrease in the percentage of cells with high potential of mitochondrial membrane in the treated group, when compared to the control, suggesting that mitochondrial damage can be the source of oxidative damage. Mitochondrial metabolism is possibly correlated to pathophysiology of oxidative homeostasis imbalance, caused by heat stress. Several studies have observed a clear relationship between sperm oxidative stress and mitochondrial activity [8, 43, 55]. Mitochondria present in sperm are the main factor responsible for ATP production by oxidative phosphorylation and thus are responsible for the production of ROS. Physiologically, about 1% of the oxygen formed is converted to superoxide anion, originating the building ROS chain [56, 57], which is fundamental to several physiological functions [58]. In the case of mitochondrial damage, ROS production may be exacerbated by the release of more prooxidative factors [55–59]. More studies contemplating mitochondrial fraction [60] and possible signaling pathways involved in mitochondrial function must be conducted to clarify how heat stress alters oxidative balance, such as cytochrome c levels by western blotting [61], disturbances in mitochondrial enzyme complexes (I–IV), and decrements in tricarboxylic acid cycle enzymes [62].

One may conclude that heat stress causes alterations in sperm during a spermatic cycle and disruption of oxidative homeostasis, due to oxidative stress, observed by increase in DCF staining and GPx enzymatic activity. This stress may possibly be caused by a mitochondrial alteration, once sperm from the ejaculate and epididymis presented a decrease in the high potential of mitochondrial membrane when the rams were submitted to heat stress. In addition, the epididymal immediate and long-term response to the heat stress do seem to be similar.

## Supplementary Material

The supplementary material contains tables describing mean, median, standard errors, quartiles and *p* value of different variables of ejaculated sperm such as motility, sperm concentration, mass motility, total defects, mayor defects, minor defects, sperm thiobarbituric acid reactive substances, seminal plasma thiobarbituric acid reactive substances, stressed cells, high mitochondrial membrane potential, low mitochondrial membrane potential, intermediate mitochondrial membrane potential, percentage of sperm cells with intact membrane and intact acrosome, percentage of sperm cells with membrane and damaged acrosome, percentage of sperm cells with damaged membrane and intact acrosome, percentage of sperm cells with damaged membrane and damaged acrosome, glutathione peroxidase enzymatic activity, glutathione reductase enzymatic activity, superoxide dismutase enzymatic activity, catalase enzymatic activity, immunodetection of catalase, immunodetection of superoxide dismutase, immunodetection of glutathione reductase, immunodetection of glutathione peroxidase considering the treatment effect between treated and control groups in rams submitted or not to heat stress.
