# Supplementary material for: Evaluation of Lasting Effects of Heat Stress on Sperm Profile and Oxidative Status of Ram Semen and Epididymal Sperm
Source: Oxid Med Cell Longev. 2016 Jan 17;2016:1687657. doi: 10.1155/2016/1687657 (PMC4737001; doi:10.1155/2016/1687657)
Supplement: Supplementary file 1 — The supplementary material contains tables describing mean, median, standard errors, quartiles and p value of different variables of ejaculated sperm such as motility, sperm concentration, mass motility, total defects, mayor defects, minor defects, sperm thiobarbituric acid reactive substances, seminal plasma thiobarbituric acid reactive substances, stressed cells, high mitochondrial membrane potential, low mitochondrial membrane potential, intermediate mitochondrial membrane potential, percentage of sperm cells with intact membrane and intact acrosome, percentage of sperm cells with membrane and damaged acrosome, percentage of sperm cells with damaged membrane and intact acrosome, percentage of sperm cells with damaged membrane and damaged acrosome, glutathione peroxidase enzymatic activity, glutathione reductase enzymatic activity, superoxide dismutase enzymatic activity, catalase enzymatic activity, immunodetection of catalase, immunodetection of superoxide dismutase, immunodetection of glutathione reductase, immunodetection of glutathione peroxidase considering the treatment effect between treated and control groups in rams submitted or not to heat stress. [file 1687657.f1.docx]

**Table S1 -** Mean and median. standard errors. quartiles and *p* value of different variables of ejaculated sperm considering the treatment effect between treated and control groups in rams submitted or not to heat stress.

| **VARIABLE** | **CONTROL** | **TREATED** | ***p*** |
| --- | --- | --- | --- |
| Motility (%) | 79.90 ± 0.66 | 69.90 ± 1.38 | < 0.0001 |
| Sperm concentration (x 10^9^ / mL) | 4.78 ±2.05 | 4.87 ±1.68 | 0.4167 |
| Mass motility (0-5) | 4.05 ± 0.08 | 3.33 ± 0.10 | 0.0011 |
| Total defects (%) | 3.45 ± 0.21 | 8.66 ±1.90 | 0.1230 |
| Mayor defects (%)* | 2 (1.5; 3) | 2.5 (1.5; 4.5) | 0.019 |
| Minor defects (%)* | 1 (0.5; 1.5) | 1.25 (0.5; 3) | 0.057 |
| sperm TBARS (ng/mL) | 2.05 ± 3.42 | 8.89 ± 0.42 | 0.24 |
| seminal plasma TBARS (ng/mL) | 275.05 ± 12.13 | 263.27 ± 12.28 | 0.5998 |
| Stressed cells (stained by DCF - %) | 3.35 ± 0.60 | 5.60 ± 2.09 | 0.05 |
| High mitochondrial membrane potential (%)* | 81.95 (74.6; 87.5) | 77.75 (62.4; 85.8) | 0.026 |
| Low mitochondrial membrane potential (%) | 4.59 ± 0.95 | 6.88 ± 1.29 | 0.2631 |
| Intermediate mitochondrial membrane potential (%) | 17.46 ± 1.68 | 21.11 ± 1.80 | 0.2297 |
| IMDA sperm (%) | 3.19 ± 0.52 | 3.67 ± 0.51 | 0.2678 |
| IMIA sperm (%) | 52.95 ± 2.05 | 41.80 ± 2.06 | 0.0019 |
| DMIA sperm (%)* | 10.15 (6.8; 17) | 18.25 (11; 24.9) | 0.044 |
| DMDA sperm (%)* | 29.4 (22.6; 35.2) | 32.55 (26.7; 41.5) | < 0.0001 |
| GPx enzymatic activity (UI/ml) | 0.00096 ± 0.000070 | 0.00120 ± 0.000069 | 0.0127 |
| GDR enzymatic activity (UI/ml) | 0.000043±0.00000221 | 0.000081±0.0000048 | 0.002 |
| SOD enzymatic activity (UI/ml) | 0.000084±0.0000049 | 0.000081±0.0000041 | 0.5239 |
| Catalase enzymatic activity (UI/ml) | 0.037±0.0006 | 0.045±0.0091 | 0.8081 |
| Immunodetection of Catalase (pixels /área) | 1.62±0.20 | 1.72±0.23 | 0.48 |
| Immunodetection of SOD (x 10^11^ pixels /área) | 1.39±0.56 | 1.04±0.749 | 0.57 |
| Immunodetection of GDR (pixels /área) | 0.35±0.05 | 0.32±0.04 | 0.65 |
| Immnodetection of GPXBI (pixels /área) | 0.64±0.08 | 0.71±0.11 | 0.74 |
| Immnodetection of GPXBS (pixels /área) |  |  |  |

Legend: DCF = dchlorofluorescein. IMIA = intact membrane and intact acrosome. IMDA = intact membrane and damaged acrosome. DMIA = damaged membrane and intact acrosome. DMDA = damaged membrane and damaged acrosome. GPx = Glutathione peroxidase. GDR = Glutathione peroxidase. SOD = superoxide dismutase. GPXBI = Glutathione peroxidase inferior band. GPXBS = Glutathione peroxidase superior band. TBARS = thiobarbituric acid reactive substances.

**Table S2 -** Mean and median. standard errors. quartiles and *p* value of different variables of ejaculated sperm considering the week effect in rams submitted or not to heat stress.

| **Weeks** | **1** | **2** | **3** | **4** | **5** | **6** | **7** | **8** | **9** | **p** |
| --- | --- | --- | --- | --- | --- | --- | --- | --- | --- | --- |
| Motility (%) | 75.41  ± 2.71 | 72.08  ± 2.34 | 72.5  ± 3.71 | 73.33  ± 4.40 | 75.41  ± 2.08 | 80  ± 2.13 | 74.58  ± 1.43 | 72.5  ± 2.71 | 78.33  ± 1.42 | 0.032 |
| Sperm Concentration  (x 10^9^ / mL) | 4.88  ± 0.31 | 2.99  ± 0.30 | 5.53  ± 0.42 | 4.93  ± 0.34 | 4.98  ± 0.29 | 5.21  ± 0.50 | 5.02  ± 0.33 | 4.87  ± 0.25 | 4.94  ± 0.37 | < 0.0001 |
| Mass motility  (0-5) | 3.58  ± 0.28 | 3.5  ± 0.23 | 3.5  ± 0.28 | 3.66  ± 0.25 | 3.83  ± 0.29 | 4  ± 0.21 | 3.83  ± 0.11 | 3.58  ± 0.22 | 3.75  ± 0.13 | 0.444 |
| Total Defects  (%) | 4.25  ± 0.61 | 3.79  ± 1 | 14.58  ± 7.5 | 6.45  ± 2.58 | 5.91  ± 2.33 | 3.91  ± 1.18 | 3.37  ± 0.59 | 7  ± 1.76 | 5.25  ± 0.97 | 0.075 |
| Mayor Defects (%)* | 2.75  (1.25;3.25) | 1.25  (1;2) | 2.75  (2.25;4) | 2  (1.5;5) | 2 (1.25;3.2) | 2  (1.5; 3.25) | 1.5  (1;2.25) | 3.25 (1.75;3.75) | 2.5  (1.5;  3.2) | > 0.05 |
| Minor Defects (%)* | 1.5  (0.75; 2) | 1.5  (1; 1.75) | 1  (1; 2.25) | 1  (0.5; 2.25) | 1  (0.5; 2.5) | 0.5  (0; 1) | 1  (0.5;2.25) | 1.75  (0.75; 3) | 2.5 (0.25;  4.5) | < 0.05 |
| sperm TBARS  (ng/mL) | 1.69  ± 0.76 | 7.16  ± 0.5 | 1.64  ± 0.55 | 2.63  ± 1.27 | 1.79  ± 0.48 | 1.82  ± 0.98 | 2.62  ± 0.5 | 2.09  ± 0.45 | 2.17  ± 0.93 | < 0.0001 |
| seminal plasma TBARS (ng/mL) | 221.26 ± 18.01 | 238.97 ± 13.64 | 226.63 ± 18.69 | 363.34 ± 31.68 | 277.1± 25.89 | 263.98 ± 28.18 | 252.82 ± 22.22 | 233.32 ± 22.43 | 345.00  ±15.09 | < 0.0001 |
| Stressed cells (stained by DCF) (%) | 2.91  ± 0.65 | 2.17  ± 0.68 | 1.82  ± 0.34 | 2.77  ± 0.84 | 0.84  ± 0.18 | 2.37  ± 0.73 | 20.89  ± 8.48 | 4.00  ± 1.11 | 2.53  ± 0.56 | 0.072 |
| High mitochondrial membrane potential (%)* | 64.3  (61.55; 75.7) | 83.65 (75.35; 86.7) | 72  (48.65; 85.1) | 79.95  (71;  86.55) | 78.1  (66.8; 83.3) | 87.35 (79.45; 89.65) | 81.65  (61.65; 88.8) | 81.05  (74.25; 85.65) | 84.35 (79.4; 88.15) | < 0.05 |
| Low mitochondrial membrane potential (%) | 7.92  ± 2.17 | 4.44  ± 1.22 | 8.84  ± 3.88 | 7.19  ± 1.82 | 3.64  ± 0.73 | 2.80  ± 0.61 | 10.59  ± 4.88 | 2.72  ± 0.26 | 3.11  ± 0.37 | 0.270 |
| Intermediate mitochondrial membrane potential (%) | 25.53  ± 2.74 | 15.48  ± 1.71 | 22.85  ± 5.55 | 18.50  ± 3.59 | 21.02  ± 3.05 | 12.60  ± 1.93 | 21.39  ± 5 | 20.19  ± 4.31 | 15.94  ± 3.61 | 0.259 |
| IMDA sperm (%) | 1.59  ± 0.68 | 2.22  ± 0.59 | 2.20  ± 0.54 | 2.63  ± 0.92 | 5.61  ± 0.74 | 2.95  ± 0.80 | 7.68  ± 2.31 | 3.11  ± 0.59 | 2.50  ± 0.50 | < 0.0001 |
| IMIA sperm (%) | 40.21  ± 4.09 | 54.60  ± 3.37 | 39.25  ± 4.21 | 36.86  ± 4.77 | 37.55  ± 3.32 | 52.82  ± 5.97 | 54.13  ± 3.73 | 52.92  ± 3.52 | 58.04 ± 3.64 | < 0.0001 |
| DMIA sperm (%)* | 43.8  (33.6; 51.35) | 26.5  (24.45; 29.8) | 32.1  (28.45; 45.1) | 30.15  (25.9; 40.3) | 34.3  (31.0;37.2) | 28.65  (17.8; 41.8) | 26.55  (21.8; 31.65) | 30.45  (25.85; 35.65) | 26.1  (21.1; 33.35) | < 0.05 |
| DMDA sperm (%)* | 14.65  (10.9; 21.6) | 12.05  (8.36; 16.6) | 20.1  (11.6; 32.35) | 24.65  (16.35; 3.15) | 18.7  (12.2; 26.8) | 9.735  (6.94; 16.3) | 10.01  (6.04; 18.2) | 13.15  (8.19; 17.85) | 9.625 (6.89; 13.95) | < 0.05 |
| GPx enzymatic activity (UI/ml) | 0.001  ±0.0001 | 0.001  ±0.0001 | 0.001  ±0.0001 | 0.001  ±0.0001 | 0.0012  ±0.0001 | 0.0008  ±0.0002 | 0.00080  ±0.0002 | 0.0008  ±0.00008 | 0.001  ±0.0001 | 0.01 |
| GDR enzymatic activity (UI/ml) | 0.000004  ±0.000001 | 0.00008  ±0.00002 | 0.000056  ±0.0000048 | 0.00005  ±0.000006 | 0.000049  ±0.000008 | 0.00004  ±0.000008 | 0.000046  ±0.0000048 | 0.000049  ±0.00001 | 0.00004±  0.00001 | 0.16 |
| SOD enzymatic activity (UI/ml) | 0.00009  ±0.00001 | 0.0001  ±0.00003 | 0.00009  ±0.00001 | 0.000068  ±0.0000081 | 0.000079  ±0.000001 | 0.00007  ±0.00015 | 0.000074  ±0.0000069 | 0.00007  ±0.0000057 | 0.00007±  0.00001 | 0.007 |
| Catalase enzymatic activity (UI/ml) | 0.047  ±0.001 | 0.05  ±0.002 | 0.043  ±0.02 | 0.074  ±0.02 | 0.01  ±0.003 | 0.02  ±0.003 | 0.025  ±0.013 | 0.084  ±0.06 | 0.038  ±0.001 | 0.35 |
| Immunodetection of Catalase (pixels /área) | 1.85  ±0.14 | 2.40  ±0.26 | 2.22  ±0.22 | 2.42  ±0.20 | 0.08  ±0.008 | 1.85  ±0.14 | 0.11  ±0.008 | 1.90  ±0.18 | 2.22  ±0.22 | < 0.001 |
| Immunodetection of SOD (x 10^11^ pixels /área) | 0.0763  ±0.0035 | 0.502  ±0.027 | 0.010  ±0.0080 | 0.56  ±0.29 | 0.094  ±0.0031 | 0.65  ±0.035 | 5.88  ±3.02 | 2.48  ±1.32 | 0.59  ±0.29 | 0.056 |
| Immunodetection of GDR (pixels /área) | 0.22  ±0.03 | 0.36  ±0.13 | 0.29  ±0.06 | 0.44  ±0.11 | 0.15  ±0.09 | 0.27  ±0.08 | 0.39  ±0.11 | 0.53  ±0.12 | 0.34  ±0.09 | 0.28 |
| Immnodetection of GPXBI (pixels /área) | 0.30  ±0.02 | 0.92  ±0.18 | 0.58  ±0.24 | 1.09  ±0.21 | 0.81  ±0.15 | 0.37  ±0.07 | 1.16  ±0.03 | 0.60  ±0.18 | 0.25  ±0.05 | 0.003 |
| Immnodetection of GPXBS (pixels /área) | 0.25  ±0.04 | 0.28  ±0.03 | 0.58  ±0.02 | 0.43  ±0.14 | 0.81  ±0.15 | 0.49  ±0.08 | 1.16  ±0.03 | 0.52  ±0.04 | 0.45  ±0.09 | 0.002 |

Legend: DCF = dchlorofluorescein. IMIA = intact membrane and intact acrosome. IMDA = intact membrane and damaged acrosome. DMIA = damaged membrane and intact acrosome. DMDA = damaged membrane and damaged acrosome. GPx = Glutathione peroxidase. GDR = Glutathione peroxidase. SOD = superoxide dismutase. GPXBI = Glutathione peroxidase inferior band. GPXBS = Glutathione peroxidase superior band. TBARS = thiobarbituric acid reactive substances.

**Table S3** - Mean and median. standard errors. quartiles and *p* value of different variables of ejaculated sperm considering the interaction effect between treatment and week in rams submitted or not to heat stress.

| **WeeksSS** | **1** | **2** | **3** | **4** | **5** | **6** | **7** | **8** | **9** | ***p*** |
| --- | --- | --- | --- | --- | --- | --- | --- | --- | --- | --- |
| Motility (%) | | | | | | | | | |  |
| **Control** | 83.33 ± 1.66 | 78.33 ± 1.05 | 81.66 ± 1.05 | 80.83 ± 1.53 | 80.83 ±1.53 | 79.16 ±2.38 | 78.33 ± 1.05 | 75.83 ± 3.96 | 80.83 ± 1.53 | 0.007 |
| **Treated** | 67.5 ± 2.14 | 65.83 ± 2.71 | 63.33 ±5.10 | 65.83 ± 7.79 | 70 ± 2.23 | 80.83 ± 3.74 | 70.83 ± 1.53 | 69.16 ± 3.51 | 75.83 2.00 |  |
| Sperm Concentration (x 10^9^ spermatozoa/mL) | | | | | | | | | |  |
| **Control** | 4.25 ± 0.34 | 3.16 ± 0.57 | 6.22± 0.59 | 4.81 ± 0.55 | 4.59 ± 0.40 | 5.05 ± 0.99 | 4.55 ± 0.44 | 5.24 ± 0.45 | 5.08 ± 0.50 | 0.907 |
| **Treated** | 5.52 ± 0.39 | 2.81 ± 0.25 | 4.85 ± 0.50 | 5.04 ± 0.40 | 5.37 ± 0.36 | 5.37 ± 0.45 | 5.48 ± 0.12 | 4.51 ± 0.58 | 4.80 ± 0.07 |  |
| Mass Motility (0 – 5) | | | | | | | | | |  |
| **Control** | 4.33 ± 0.21 | 4 ± 0.36 | 4.33 ± 0.21 | 4 ± 0 | 4.5 ± 0.22 | 4 ± 0.25 | 4 ± 0 | 3.66 ± 0.42 | 3.66 ± 0.21 | 0.000 |
| **Treated** | 2.83 ± 0.30 | 3 ± 0 | 2.66 ± 0.21 | 3.33 ± 0.49 | 3.16 ± 0.40 | 4 ± 0.36 | 3.66 ± 0.21 | 3.5 ± 0.22 | 3.83 ± 0.16 |  |
| Total Defects (%) | | | | | | | | | |  |
| **Control** | 3.41 ± 0.41 | 3.33± 0.27 | 3.75 ± 0.55 | 2.91 ± 0.74 | 3.41 ± 0.67 | 3.16 ± 0.87 | 2.83 ± 0.27 | 3.83 ± 0.57 | 4.41 ± 1.04 | 0.338 |
| **Treated** | 5.08 ± 1.09 | 4.25 ± 2.06 | 25.41 ± 4.30 | 10 ± 4.88 | 8.41 ± 4.59 | 4.66 ± 2.28 | 3.91 ± 1.17 | 10.16 ± 3.06 | 6.08 ± 1.69 |  |
| Mayor Defects (%)* | | | | | | | | | | |
| **Control** | 1.75 (1 ; 2.5) | 1.5 (1.5; 2.5) | 2.75 (2.5; 3.5) | 2 (1;3) | 1.5 (1;2.5) | 2.25 (1.5;3) | 1.25 (1; 1.5) | 2.75 (1.5; 3.5) | 2.5 (2.5; 3.5) | > 0.05 |
| **Treated** | 3 (3;3.5) | 1 (1;1) | 3 (2;2) | 2 (2;7) | 3.25 (1.5; 6.5) | 2 (1.5; 3) | 2.25 (1.5; 4.5) | 3.5 (2;4) | 2.25 (1;3) |  |
| MInor Defects (%)* | | | | | | | | | | |
| **Control** | 1.5 (1;2) | 1.5 (1.5; 1.5) | 1 (0.5; 1) | 0.75 (0;1) | 1.5 (0.5; 2.5) | 0.5 (0;1) | 1.5 (1; 2.5) | 0.75 (0.5; 2.5) | 1.75 (0;3) | < 0.05 |
| **Treated** | 1.5 (0.5;2) | 1.25 (1;2.5) | 2 (1;31) | 2.25 (0.5; 3.5) | 0.5 (0.5; 2.5) | 0.5 (0;1) | 0.5 (0.5;2) | 2.25 (2; 5.5) | 3.25 (1; 5.5) |  |
| Sperm TBARS (ng/mL) | | | | | | | | | |  |
| **Control** | 1.99 ± 0.99 | 3.63 ± 1.44 | 1.59 ± 0.60 | 2.16 ± 1.57 | 1.87 ± 0.45 | 2.41 ± 1.10 | 1.97 ± 0.72 | 1.92 ± 0.36 | 2.13 ± 0.81 | 0.170 |
| **Treated** | 1.38 ± 0.25 | 10.67±10.69 | 1.76 ± 0.51 | 3.10 ± 0.75 | 1.70 ± 0.53 | 1.24 ± 0.27 | 3.26 ± 3.84 | 2.25 ± 0.49 | 2.20 ± 1.12 |  |
| seminal plasma TBARS (ng/mL) | | | | | | | | | |  |
| **Control** | 238.81 ± 33.20 | 245.41 ± 15.66 | 197.36 ± 11.16 | 350.68 ± 20.83 | 300.93 ± 49.07 | 310.7 ± 46.22 | 259.21 ± 29.41 | 217.86 ± 28.65 | 354.46 ± 27.75 | 0.338 |
| **Treated** | 203.71 ± 14.69 | 232.56 ± 23.59 | 255.9 ± 2.73 | 376 ± 62.60 | 253.26 ± 17.75 | 217.26 ± 22.05 | 246.43 ± 35.93 | 248.78 ± 36.01 | 335.55 ± 14.02 |  |
| Stressed cells (stained by DCF)  **(%)** | | | | | | | | | |  |
| **Control** | 2.64 ± 1.07 | 1.28 ± 0.46 | 2.06 ± 0.64 | 3.35 ± 1.48 | 0.67 ± 0.18 | 1.84 ± 0.60 | 8.73 ± 4.14 | 6.72 ± 1.54 | 2.87 ± 0.36 | 0.010 |
| **Treated** | 3.18 ± 0.82 | 3.05 ± 1.23 | 1.57 ± 0.30 | 2.19 ± 0.89 | 1.02 ± 0.32 | 2.89 ± 1.37 | 33.05 ± 5.51 | 1.27 ± 0.38 | 2.20 ± 1.09 |  |
| High mitochondrial membrane potential (%)* | | | | | | | | | | |
| **Control** | 67.25  (62.8; 80.2) | 84.2  (80;86.6) | 82.2  (74.9; 91.1) | 77.55  (71.3; 86) | 81  (75.5; 82.1) | 88.6  (85.1; 93.1) | 86.85  (60.9; 92.5) | 82.55  (76.8; 87.3) | 81.4  (76.1; 89.6) | < 0.05 |
| **Treated** | 62.2  (61.2; 75.1) | 80.45  (74.5; 86.8) | 61.55  (24.8; 69.1) | 82.25  (41.6; 88.9) | 68.5  (56.9; 87.6) | 82.35  (72.8; 87.5) | 79.65  (62.4; 82) | 79.45  (71.7; 84) | 84.35  (83.5; 86.9) |  |
| Low mitochondrial membrane potential (%)* | | | | | | | | | |  |
| **Control** | 5.85 ± 1.78 | 4.28 ± 1.76 | 3.23 ± 1.10 | 6.46 ± 1.72 | 3.34 ± 1.01 | 2.57 ± 1.05 | 9.81 ± 8.04 | 2.59 ± 0.27 | 3.23 ± 0.63 | 0.766 |
| **Treated** | 10.02 ± 3.98 | 4.6 ± 1.85 | 14.46 ± 7.25 | 7.93 ± 3.38 | 3.95 ± 1.15 | 3.03 ± 0.75 | 11.37 ± 6.31 | 2.88 ± 0.50 | 2.99 ± 0.63 |  |
| Intermediate mitochondrial membrane potential (%)* | | | | | | | | | |  |
| **Control** | 24.88 ± 4.97 | 15.61 ± 3.13 | 19.16 ± 8.61 | 15.17 ± 1.83 | 17.3 ± 2.25 | 9.08 ± 1.09 | 19.96 ± 7.81 | 16.10 ± 3.44 | 19.92 ± 7.11 | 0.871 |
| **Treated** | 26.18 ± 2.89 | 15.35 ± 1.76 | 26.54 ± 7.49 | 21.83 ± 7.00 | 24.75 ± 5.52 | 16.13 ± 3.20 | 22.82 ± 6.93 | 25.09 ± 8.56 | 11.96 ± 0.79 |  |
| IMDA sperm (%) | | | | | | | | | |  |
| **Control** | 1.06 ± 0.38 | 2.79 ± 1.06 | 1.36 ± 0.34 | 1.40 ± 0.42 | 6.39 ± 1.08 | 3.29 ± 1.57 | 8.01 ± 3.38 | 2.31 ± 0.25 | 2.15 ± 0.68 | 0.339 |
| **Treated** | 2.12 ± 1.34 | 1.64 ± 0.52 | 3.04 ± 0.96 | 4.71 ± 1.56 | 4.83 ± 1.01 | 2.61 ± 0.55 | 7.36 ± 3.48 | 3.90 ± 1.11 | 2.85 ± 0.77 |  |
| IMIA sperm (%) | | | | | | | | | |  |
| **Control** | 38.08 ± 4.37 | 58.06 ± 5.52 | 48.76 ± 2.90 | 44.43 ±5.82 | 41.166±5.37 | 65.38 ± 6.59 | 53.58 ± 5.58 | 60.96 ± 5.06 | 66.18 ± 2.67 | 0.028o |
| **Treated** | 42.35 ± 7.26 | 51.15 ± 3.86 | 29.73 ± 5.78 | 29.29 ± 6.58 | 33.95 ± 3.81 | 40.26 ± 7.12 | 54.68 ± 5.48 | 44.88 ± 1.76 | 49.9 ± 4.98 |  |
| DMIA sperm (%)* | | | | | | | | | | |
| **Control** | 42.05  (34.2; 58.9) | 25  (24;27.5) | 36.95  ( 30.2; 46.6) | 33.1  (27.1; 45.1) | 33.25  (29.7; 34.4) | 17.8  (13.3; 30) | 24.35  (21.6; 28) | 25.85  (22; 29.3) | 21.9  (20.8; 22.6) | < 0.05 |
| **Treated** | 45.85  (23.1; 48.4) | 29.4  ( 25.5; 41.5) | 32.05  (26.7; 43.6) | 27.95  (20.9; 39.4) | 36.45  (32; 51.2) | 39.4  (27.3; 43.7) | 27.85  (23.8; 34) | 32  (30.9; 40.7) | 33.15  (29.6; 37) |  |
| DMDA sperm (%)* | | | | | | | | | | |
| **Control** | 12.3  (11.3; 21.7) | 9.66  (6.82. 14.9) | 11.6  (5.62; 14.6) | 19.85  (10.5; 22.9) | 17.2  (11.9; 29.2) | 6.94  (5.75; 7.77) | 9.38  (6.5; 13.8) | 8.195  (6.5; 13.8) | 7.595  (3.14; 9.86) | < 0.05 |
| **Treated** | 17.25  (10.5; 21.5) | 14.05  (10; 17.6) | 32.35  ( 21.6; 36.7) | 33.15  (28.4; 43.7) | 19.3  (12.5; 31.9) | 16.3  (12.1; 31.9) | 13.56  (5.41; 19.2) | 15.35  (12.8; 18.5) | 10.75  (9.39; 22) |  |
| GPx enzymatic activity (UI/ml) | | | | | | | | | | |
| **Control** | . | . | 0.0010397 | 0.0013934 | . | 0.001179 | 0.000610932 | 0.000889603 | 0.000889603 | 0.90 |
| **Treated** | 0.0014898 | 0.0011254 | 0.0012755 | 0.0011683 | 0.0012433 | 0.0016613 | 0.0010289 | 0.000728832 | 0.0012433 |  |
| GDR enzymatic activity (UI/ml) | | | | | | | | | | |
| **Control** | 0.000048 | 0.000054662 | 0.000051447 | 0.000041801 | 0.000041801 | 0.000038585 | 0.000041801 | 0.00003537 | 0.000045016 | 0.75 |
| **Treated** | 0.000045016 | 0.000109325 | 0.000061093 | 0.000061093 | 0.000057878 | 0.000054662 | 0.000051447 | 0.000064309 | 0.000051447 |  |
| SOD enzymatic activity (UI/ml) | | | | | | | | | | |
| **Control** | 0.000076389 | 0.000134259 | 0.000108796 | 0.000076389 | 0.000078704 | 0.00005787 | 0.00006713 | 0.000083333 | 0.000074074 | 0.56 |
| **Treated** | 0.000106481 | 0.00006713 | 0.000085648 | 0.000060185 | 0.000081019 | 0.000087963 | 0.000081019 | 0.000071759 | 0.000071759 |  |
| Catalase enzymatic activity (UI/ml) **(UI/ml)** | | | | | | | | | | |
| **Control** | 0.0458716 | 0.0527523 | 0.0229358 | 0.0481651 | 0.0068807 | 0.0321101 | 0.0389908 | 0.0183486 | 0.0389908 | 0.42 |
| **Treated** | 0.0481651 | 0.0573394 | 0.0642202 | 0.1009174 | 0.0137615 | 0.0252294 | 0.0114679 | 0.1513761 | . |  |
| Immunodetection of Catalase (pixels /área) | | | | | | | | | | |
| **Control** | 1.63±0.14 | 2.47±0.007 | 2.15±0.04 | 2.37±0.32 | 0.09±0.01 | 1.63±0.14 | 0.12±0.01 | 2.00±0.07 | 2.15±0.04 | 0.95 |
| **Treated** | 2.07±0.10 | 2.34±0.64 | 2.29±0.55 | 2.48±0.35 | 0.077±0.01 | 2.07±0.10 | 0.10±0.01 | 1.79±0.42 | 2.29±0.55 |  |
| Immunodetection of SOD (x 10^11^ pixels /área) | | | | | | | | | | |
| **Control** | 0.011±0.05 | 0.59±0.5 | 0.014±0.014 | 1.03±0.25 | 0.12±0.02 | 0.71±0.71 | 4.81±4.10 | 4.77±0.15 | 4.23±0.41 | 0.37 |
| **Treated** | 0.038±0.0381 | 0.43±0.41 | 0.0019±0.0014 | 0.093±0.0016 | 0.006±0.0061 | 0.60±0.48 | 6.96±5.96 | 0.20±0.13 | 0.944±0.01 |  |
| Immunodetection of GDR (pixels /área) | | | | | | | | | | |
| **Control** | 0.26±0.04 | 0.27±0.16 | 0.40±0003 | 0.31±0.13 | 0.26±0.18 | 0.25±0.19 | 0.41±0.26 | 0.63±0.27 | 0.34±0.20 | 0.66 |
| **Treated** | 0.18±0.06 | 0.45±0.25 | 0.19±0.03 | 0.56±0.18 | 0.037±0.018 | 0.29±0.05 | 0.36±0.13 | 0.43±0.06 | 0.35±0.06 |  |
| Immnodetection of GPXBI (pixels /área) | | | | | | | | | | |
| **Control** | 0.28±0.02 | 0.73±0.04 | 0.41±0.008 | 1.13±0.39 | 0.91±0.001 | 0.47±0.05 | 1.11±0.004 | 0.47±0.19 | 0.23±0.10 | 0.88 |
| **Treated** | 0.33±0.03 | 1.11±0.34 | 0.76±0.53 | 1.04±0.35 | 0.713±0.35 | 0.27±0.10 | 1.20±0.06 | 0.73±0.37 | 0.27±0.06 |  |
| Immnodetection of GPXBS (pixels /área) | | | | | | | | | | |
| **Control** | 0.18±0.01 | 0.30±0.07 | 0.57±0.006 | 0.43±0.32 | 0.91±0.01 | 0.12±0.01 | 1.11±0.004 | 0.53±0.07 | 0.37±0.04 | 0.87 |
| **Treated** | 0.31±0.06 | 0.26±0.0004 | 0.60±0.062 | 0.43±0.13 | 0.712±0.35 | 0.42±0.12 | 1.20±0.06 | 0.51±0.06 | 0.53±0.20 |  |

Legend: DCF = dchlorofluorescein. IMIA = intact membrane and intact acrosome. IMDA = intact membrane and damaged acrosome. DMIA = damaged membrane and intact acrosome. DMDA = damaged membrane and damaged acrosome. GPx = Glutathione peroxidase. GDR = Glutathione peroxidase. SOD = superoxide dismutase. GPXBI = Glutathione peroxidase inferior band. GPXBS = Glutathione peroxidase superior band. TBARS = thiobarbituric acid reactive substances.

**Table S4 -** Mean. standard errors and *p* value of different variables of epididymal sperm considering the treatment effect between treated and control groups in rams submitted or not to heat stress.

| **Variables** | **Control** | **Treated** | ***p*** | |
| --- | --- | --- | --- | --- |
| IMIA sperm (%) | 80.50±2.12 | 78.46±4.46 | 0.58 | |
| IMDA sperm (%) | 1.43±0.30 | 1.78±0.35 | 0.47 | |
| DMDA sperm (%) | 4.72±0.70 | 5.17±1.03 | 0.96 | |
| DMIA sperm (%) | 13.33±1.50 | 12.453±2.58 | 0.47 | |
| High mitochondrial membrane potential (%) | 78.39±2.29 | 61±5.62 | 0.002 | |
| Low mitochondrial membrane potential (%) | 4.15±1.35 | 9.044±2.06 | 0.02 | |
| Intermediate mitochondrial membrane potential (%) | 18.41±1.45 | 29.96±4.71 | | 0.01 |
| Stressed cells (stained by DCF) | 1.10±0.33 | 2.27±0.56 | 0.03 | |
| sperm TBARS (ng/mL) | 854.05±74.28 | 813.53±26.92 | | 0.87 |
| Average velocity (VAP. μm/s) | 148.141±9.83 | 139±10.73 | 0.55 | |
| Straight-line velocity (VSL. μm/s) | 117.61±9.27 | 112.18±9.75 | 0.69 | |
| Curvilinear velocity (VCL. μm/s) | 236.625±11.43 | 217.4±12.96 | 0.30 | |
| Amplitude of lateral head displacement (ALH. μm/s) | 8.025±0.32 | 7.62±0.34 | 0.44 | |
| Beating cross frequency (BCF. Hz) | 41.391±1.00 | 40.01±0.70 | 0.29 | |
| Straightness coefficient (STR. %) | 76.5±2.18 | 77.3±1.38 | 0.77 | |
| Linearity coefficient (LIN. %) | 49.5±2.89 | 51.3±2.13 | 0.52 | |
| Total motility (%) | 78.66±4.12 | 72.9±6.70 | 0.49 | |
| Progressive motility (%) | 43.083±4.12 | 40±3.69 | 0.59 | |
| Fast movement sperm (%) | 69.16±4.57 | 62.7±6.72 | 0.43 | |
| Medium movement sperm (%) | 9.5±1.10 | 10.3±1.57 | 0.82 | |
| Slow movement sperm (%) | 7.33±1.15 | 6±0.77 | 0.36 | |
| Static movement sperm (%) | 13.83±3.95 | 21.2±6.52 | 0.48 | |
| Enzymatic activity of GPx (UI/ml) | 14.38±3.04 | 16.18±2.26 | 0.37 | |
| Enzymatic activity of SOD (UI/ml) | 121.35±12.97 | 98.58±12.89 | 0.22 | |

Legend: DCF = dchlorofluorescein. IMIA = intact membrane and intact acrosome. IMDA = intact membrane and damaged acrosome. DMIA = damaged membrane and intact acrosome. DMDA = damaged membrane and damaged acrosome. GPx = Glutathione peroxidase. SOD = superoxide dismutase. TBARS = thiobarbituric acid reactive substances.

**Table S5 -** Mean. standard errors and *p* value of different variables of epididymal sperm considering the immediate and long-term response in rams submitted or not to heat stress.

| **Variables** | **Immediate response** | **Long-term response** | ***p*** |
| --- | --- | --- | --- |
| IMIA sperm (%) | 75.26±3.21 | 83.44±3.13 | 0.08 |
| IMDA sperm (%) | 1.27±0.31 | 1.94±0.32 | 0.18 |
| DMDA sperm (%) | 5.90±0.82 | 3.99±0.85 | 0.27 |
| DMIA sperm (%) | 15.70±1.45 | 10.63±2.08 | 0.18 |
| High mitochondrial membrane potential (%) | 61.2±5.66 | 76.75±3.44 | 0.06 |
| Low mitochondrial membrane potential (%) | 10.44±1.76 | 2.75±1.16 | 0.0001 |
| Intermediate mitochondrial membrane potential (%) | 28.76±5.00 | 20.48±2.44 | 0.06 |
| Stressed cells (stained by DCF) | 2.091±0.61 | 1.27±0.32 | 0.38 |
| sperm TBARS (ng/mL) | 820.75±46.8 | 846.83±63.93 | 0.83 |
| Average velocity (VAP. μm/s) | 147.95±5.77 | 140.018±12.59 | 0.45 |
| Straight-line velocity (VSL. μm/s) | 118.82±5.77 | 111.46±12.10 | 0.41 |
| Curvilinear velocity (VCL. μm/s) | 228.27±9.38 | 227.5±14.98 | 0.30 |
| Amplitude of lateral head displacement (ALH. μm/s) | 7.92±0.31 | 7.75±0.36 | 0.44 |
| Beating cross frequency (BCF. Hz) | 41.7±0.75 | 39.82±0.98 | 0.16 |
| Straightness coefficient (STR. %) | 77.09±1.34 | 76.63±2.35 | 0.95 |
| Linearity coefficient (LIN. %) | 51.72±1.89 | 48.90±3.15 | 0.40 |
| Total motility (%) | 77.63±3.41 | 74.45±7.27 | 0.74 |
| Progressive motility (%) | 43.27±3.24 | 40.09±4.59 | 0.65 |
| Fast movement sperm (%) | 66.09±3.05 | 66.36±7.40 | 0.91 |
| Medium movement sperm (%) | 11.63±1.35 | 8.09±1.03 | 0.02 |
| Slow movement sperm (%) | 7.72±0.88 | 5.72±1.09 | 0.07 |
| Static movement sperm (%) | 14.36±3.24 | 20±6.65 | 0.76 |
| Enzymatic activity of GPX (UI/ml) | 15±2.66 | 15.56±2.72 | 0.93 |
| Enzymatic activity of SOD (UI/ml) | 96.16±13.34 | 123.77±12.05 | 0.14 |

Legend: DCF = dchlorofluorescein. IMIA = intact membrane and intact acrosome. IMDA = intact membrane and damaged acrosome. DMIA = damaged membrane and intact acrosome. DMDA = damaged membrane and damaged acrosome. GPx = Glutathione peroxidase. SOD = superoxide dismutase. TBARS = thiobarbituric acid reactive substances.

**TABLE S6 -** Mean. standard errors and *p* value of different variables of epididymal sperm considering the interaction effect between treatments and immediate and long-term response in rams submitted or not to heat stress.

| **Treatment** | **Immediate response** | **Long-term response** | ***p*** |
| --- | --- | --- | --- |
| IMIA sperm (%) | | | |
| Controle | 77.9±1.100 | 83.116±3.996 | 0.49 |
| Tratado | 72.1±7.093 | 83.766±5.224 |  |
| IMDA sperm (%) | | | |
| Controle | 1.123±0.25 | 1.744±0.56 | 0.60 |
| Tratado | 1.425±0.60 | 2.141±0.37 |  |
| DMDA sperm (%) | | | |
| Controle | 5.131±0.7616 | 4.308±1.2365 | 0.38 |
| Tratado | 6.676±1.480 | 3.681±1.2917 |  |
| DMIA sperm (%) | | | |
| Controle | 15.816±1.418 | 10.86±2.355 | 0.70 |
| Tratado | 15.53±3.297 | 10.401±3.689 |  |
| High mitochondrial membrane potential (%) | | | |
| Controle | 72.92±3.15 | 82.95±1.87 | 0.61 |
| Tratado | 51.43±8.31 | 70.56±5.78 |  |
| Low mitochondrial membrane potential (%) | | | |
| Controle | 7.348±1.965 | 0.97±0.305 | 0.64 |
| Tratado | 13.54±2.457 | 4.548±2.13 |  |
| Intermediate mitochondrial membrane potential (%) | | | |
| Controle | 21.24±1.87 | 16.06±1.73 | 0.96 |
| Tratado | 35.03±8.48 | 24.9±3.93 |  |
| Stressed cells (stained by DCF) | | | |
| Controle | 1.50±0.61 | 0.70±0.24 | 0.82 |
| Tratado | 2.79±1.12 | 1.83±0.51 |  |
| sperm TBARS (ng/mL) | | | |
| Controle | 833.93±86.43 | 874.18±129.01 | 0.98 |
| Tratado | 807.58±46.16 | 819.48±32.31 |  |
| Average velocity (VAP. μm/s) | | | |
| Controle | 154.48±9.85 | 141.8±17.66 | 0.76 |
| Tratado | 140.12±10.74 | 137.88±20.06 |  |
| Straight-line velocity (VSL. μm/s) | | | |
| Controle | 124.53±7.19 | 110.7±17.53 | 0.59 |
| Tratado | 111.98±9.16 | 112.38±18.54 |  |
| Curvilinear velocity (VCL. μm/s) | | | |
| Controle | 235.68±13.41 | 237.56±19.86 | 0.87 |
| Tratado | 219.38±13.35 | 215.42±24.01 |  |
| Amplitude of lateral head displacement (ALH. μm/s) | | | |
| Controle | 7.93±0.53 | 8.11±0.42 | 0.39 |
| Tratado | 7.92±0.34 | 7.32±0.60 |  |
| Beating cross frequency (BCF. Hz) | | | |
| Controle | 42.53±1.28 | 40.25±1.51 | 0.72 |
| Tratado | 40.7±0.42 | 39.32±1.33 |  |
| Straightness coefficient (STR. %) | | | |
| Controle | 78.16±2.19 | 74.83±3.89 | 0.26 |
| Tratado | 75.8±1.39 | 78.8±2.35 |  |
| Linearity coefficient (LIN. %) | | | |
| Controle | 52.83±3.12 | 46.16±4.75 | 0.23 |
| Tratado | 50.4±2.06 | 52.2±3.99 |  |
| Total motility (%) | | | |
| Controle | 82.166±3.57 | 75.16±8.94 | 0.62 |
| Tratado | 72.2±5.62 | 73.6±13.05 |  |
| Progressive motility (%) | | | |
| Controle | 47.66±4.60 | 38.5±6.74 | 0.26 |
| Tratado | 38±3.64 | 42±6.78 |  |
| Fast movement sperm (%) | | | |
| Controle | 72.33±2.62 | 66±9.01 | 0.38 |
| Tratado | 58.6±3.90 | 66.8±13.41 |  |
| Medium movement sperm (%) | | | |
| Controle | 9.83±1.62 | 9.16±1.64 | 0.08 |
| Tratado | 13.8±1.98 | 6.8±1.06 |  |
| Slow movement sperm (%) | | | |
| Controle | 7.5±1.64 | 7.16±1.77 | 0.13 |
| Tratado | 8±0.44 | 4±0.70 |  |
| Static movement sperm (%) | | | |
| Controle | 9.66±2.64 | 18±7.41 | 0.46 |
| Tratado | 20±5.72 | 22.4±12.58 |  |
| Enzymatic activity of GPX (UI/ml) | | | |
| Controle | 15.21±4.53 | 14.79±3.24 | 0.51 |
| Tratado | 13.55±4.45 | 17.57±3.34 |  |
| Enzymatic activity of SOD (UI/ml) | | | |
| Controle | 110.26±19.41 | 82.06±18.08 | 0.76 |
| Tratado | 132.44±17.73 | 115.10±17.17 |  |

Legend: DCF = dchlorofluorescein. IMIA = intact membrane and intact acrosome. IMDA = intact membrane and damaged acrosome. DMIA = damaged membrane and intact acrosome. DMDA = damaged membrane and damaged acrosome. GPx = Glutathione peroxidase. SOD = superoxide dismutase. TBARS = thiobarbituric acid reactive substances.
